# Early recognition of sudden cardiac arrest in athletes during sports activity

**DOI:** 10.1007/s12471-017-1061-5

**Published:** 2017-12-01

**Authors:** N. M. Panhuyzen-Goedkoop, H. J. Wellens, J. J. Piek

**Affiliations:** 1 AMC Heart Center, Academic Medical Center, Amsterdam, The Netherlands; 2 Sports Medical Center Papendal Arnhem, Arnhem, The Netherlands; 3 Radboud University Medical Center, Nijmegen, The Netherlands; 4 Cardiovascular Research Center, Maastricht, The Netherlands

**Keywords:** Athlete, Sudden cardiac arrest, Resuscitation, AED, Syncope, Collapse

## Abstract

**Introduction:**

Sudden cardiac arrest (SCA) in athletes is an unexpected life-threatening event, which is often not recognised early and cardiopulmonary resuscitation (CPR) is not always initiated immediately. We describe key features to rapidly recognise non-traumatic SCA in athletes during sports activity.

**Methods:**

We reviewed videos and images of athletes suffering from non-traumatic SCA during sports activity. We searched Google images, Google videos and YouTube.com using the keywords ‘sudden cardiac death athlete’ and ‘resuscitation athlete’. We analysed (1) the athlete’s performance before syncope, (2) the athlete’s performance at the start of syncope, (3) the position of the body, and (4) the athlete’s facial expressions before CPR. We analysed our data by describing these four features to answer our research question.

**Results:**

We analysed the sequence of events in six well-known soccer players in whom a camera-witnessed non-traumatic SCA occurred during their athletic activity. All six athletes showed no changes before syncope. Four became unstable while standing and unexpectedly collapsed falling on their back. Two suddenly ‘dropped dead’ and fell face down. All six had their eyes wide open with a fixed gaze and fixed pupils.

**Conclusions:**

Sudden unexpected loss of consciousness in an athlete in action and a fixed gaze eye position are key features of SCA. Immediate cardiac massage should follow. The described features to immediately recognise SCA in athletes during sports activity should be taught to everyone involved in athletic activity leading to earlier recognition of SCA followed by earlier CPR.

## Introduction

Sudden cardiac arrest (SCA) in athletes in action is an unexpected and life-threatening event. This non-traumatic syncope is almost always caused by a lethal cardiac arrhythmia—i. e. ventricular tachycardia/fibrillation. Immediate bystander cardiopulmonary resuscitation (CPR) and on-site defibrillation with an automated external defibrillator (AED) should be initiated to save the athlete’s life [1–11]. If ventricular fibrillation is not terminated with defibrillation the probability of surviving will decline by 10% per minute of ongoing ventricular fibrillation [3, 5, 10]. However, a non-traumatic syncope in athletes during sports activity is usually not immediately recognised as a circulatory arrest and time is waisted by checking breathing and body movement [2, 4, 10, 12]. Unfortunately, athletes still die or develop serious cerebral damage because immediate CPR procedures, with the emphasis on cardiac massage, are not initiated immediately, even nowadays. The reason being that the lethal danger of SCA in a non-responding and non-breathing athlete is not recognised immediately [2]. Early recognition is a key feature and the bystander(s) should not hesitate to start cardiac massage immediately to restore the body circulation [4, 10, 13]. Time should not be waisted by checking and opening the airway, because in the alarming situation of an SCA the victim will not breath [12].

This article reviews what can be observed during a non-traumatic syncope in athletes during sports activity and how we can recognise sudden cardiac arrest, emphasising the importance of starting CPR immediately to restore circulation.

## Methods

We have reviewed videos and images—posted on the internet—of athletes suffering from a non-traumatic SCA during sports activity to help recognise SCA in athletes. We searched for Google images, Google videos and YouTube.com using the combined keywords ‘sudden cardiac death athlete’ and ‘resuscitation athlete’. We analysed in six well-known elite soccer players (1) the behaviour of the athlete shortly prior to syncope, (2) the behaviour of the athlete at the start of syncope, (3) the ‘dropping dead’ position of the athlete, and (4) the facial expression—with special attention to the eyes and pupils—of the athlete before receiving cardiac massage or an electrical shock.

To answer our research question of how we can better recognise non-traumatic SCA in athletes during sports activities we analysed our data by describing these four key features.

## Results

As our search on the internet could not provide a complete cohort of camera-witnessed non-traumatic SCAs in athletes, we decided to use some well-known SCA/SCD examples in elite soccer players—MVF (died 2003), MF (died 2004), AP (died 2007), FM (SCA 2012), PM (died 2012), AN (SCA 2017)—in an effort to answer the question of how we can recognise non-traumatic SCA in athletes early on.

We observed that all six well-known elite soccer players were actively engaged in the match without trauma or bodily collision prior to syncope (Table 1). Body behaviour at the time of syncope was not uniform (Table 1). Two athletes were leaning forward with their heads bent down, followed by collapsing to a sitting position. One athlete collapsed to his knees and attempted several times to get up. One athlete was walking to the side of the field and tried to sit down. All four athletes finally dropped down on their back with such a force that they bounced their upper torso and head before lying flat with their arms spread out or alongside their body (Table 1). The two remaining athletes suddenly ‘dropped dead’, falling face down without any movement afterwards. All six athletes had their eyes wide open with fixed pupils (Table 1). In none of these athletes an immediate cardiac massage was initiated. In two of the six players there was no immediate loss of conscience, suggesting an episode of ventricular tachycardia before ventricular fibrillation. Autopsy in one of these two showed arrhythmogenic right ventricular cardiomyopathy.

**Table 1:** How to recognise SCA in athletes on the field; six examples of elite soccer players

Athlete, age (year of SCA)	Performance of the athlete prior to syncope	Initiation of syncope	‘Dropping dead’ position	Eyes and position of the pupils	Cause of SCA and outcome
*MVF, 28 yrs. (2003)*	Participating in the game	Collapsing landing face down	Lying face down	Eyes wide open with fixed pupils	HCM, died
*MF, 25 yrs. (2004)*	Participating in the game	Grabbing his head, bending forward with his arms on his knees, falling left-backwards, landing on his back, and bouncing	Lying on his back, arms wide	Eyes wide open with fixed pupils	HCM, died
*AP, 22 yrs. (2007)*	Participating in the game	Grabbing his head, bending forward, collapsing to his knees	Without resuscitation spontaneous recovery, and sitting upright while being checked	in sitting upright position, eyes wide open with fixed pupils	ARVC, died
*FM, 24 yrs. (2012)*	Participating in the game	Collapsing landing face down	Lying face down, arms alongside his body with forearms underneath his body	Eyes wide open with fixed pupils	HCM (?), survived with complete recovery, ICD implanted, disqualified
*PM, 25 yrs. (2012)*	Participating in the game	Collapsing to his knees, trying to get up, but falling 2 × to his knees again, finally falling forward landing face down	Lying face down, arms bent partly under his shoulders	Eyes wide open with fixed pupils	ARVC, died
*AN, 20 yrs. (2017)*	Participating in the game	Trying to walk to the side of the field, trying to sit down, falling on his back and bouncing, trying to lift his arms	Lying on his back, arms alongside his body	Eyes wide open with fixed pupils	Cardiac arrest, severe and permanent cerebral damage

*ARVC* arrhythmogenic right ventricular cardiomyopathy*, HCM* hypertrophic cardiomyopathy, *ICD* implantable cardioverter defibrillator, *SCA* sudden cardiac arrest

## Discussion

Sudden cardiac arrest or death (SCA/SCD) in athletes during sports activity remains one of the most horrifying and tragic events. The impact of this unforgettable and unexpected lethal event in a seemingly healthy athlete is huge for everyone involved—coaches, teammates, paramedical and medical personnel, family, spectators. Also, the media coverage it receives is intense with media showing, even many years later, images and videos of an athlete’s SCA/SCD. These can easily be found on the internet. Out of 100,000 athletes with an SCA per year worldwide 0.6–2.85 athletes die [14–17]. Some of these deaths can be prevented by screening for eligibility, treating the condition and disqualifying athletes at risk of SCA/SCD from sports participation [2–4, 14–17]. The survival rates of SCA in student-athletes can be high (89%) through early recognition with bystander CPR and AED defibrillation, as was observed in a two-year prospective study in 2,149 high schools equipped with an AED [18]. During the study period, 59 cases of SCA were reported. Of these cases, 18 student-athletes received immediate CPR on the field and 16 (89%) of them survived [18]. In a German registry (2012–2014) of sports-related SCDs, Bohm et al. found that many sports-related SCAs were witnessed in public sports facilities (87%) and immediate CPR was initiated in 82% [9]. In 48 out of 55 cases, the initial rhythm was ventricular fibrillation [9]. In a similar French registry (2005–2010), Marijon et al. observed that most SCAs in athletic activity were witnessed but that bystander defibrillation with an AED was extremely rare (<1%), even though the initial rhythm was ventricular fibrillation (58.8%) [8]. The survival rate was higher in sports participants (22.8%) compared with the overall population (8%) [8]. In the Dutch ARREST study (2005–2009) of the province of North Holland, Berdowski et al. found that continuation of bystander CPR, including the use of an AED, resulted in a higher survival rate (41%, 131/320) compared with ambulance paramedics taking over the CPR procedure from the bystanders (37%, 41/110) [6]. The initial rhythm was ventricular fibrillation in 98% (108 cases) and ventricular tachycardia in 2 cases [6]. In certain cases, SCA during sports activity is the first symptom of an underlying cardiac disorder, i. e. inherited cardiac disease—hypertrophic cardiomyopathy, channelopathy – a coronary anomaly — or viral myocarditis in athletes age 35 and younger [8–10, 15, 16]. In traumatic syncope the possibility of a cardiac concussion due to blunt chest trauma inducing asystole or ventricular fibrillation must be considered [4, 9, 15, 19].

What do we learn from all these camera-witnessed SCA/SCD cases in athletes during their sports activity? Why do athletes still die on the field despite updated CPR protocols, teaching courses and the use of an AED, all of which were shown to improve the chance of survival [1, 4, 6, 8, 9, 13, 18, 20, 21]? The crucial factor is time lost because bystanders do not expect SCA in a healthy athlete resulting in a delay of cardiac massage to ensure brain perfusion and coronary perfusion. The cardiac massage should be followed by the next step in the chain of survival—i. e. early defibrillation [2, 4, 8–10, 13, 18, 20–22]. Resuscitation councils have published information on immediate bystander CPR using the protocol of airway-breathing-circulation (ABC) and restoring the heart rhythm using an AED [4, 13]. The European Society of Cardiology (ESC) Section Sports Cardiology, the American Heart Association (AHA), the American College of Cardiology (ACC), and the American National Collegiate Athletic Association (NCAA) have provided detailed medical action plans in case of cardiac events during athletic activity—training personnel in basic life support (BLS), use of an AED, mobile communication systems, dialling 112 (EU) or 911 (US) [4, 10, 23]. Also, the international sports governing bodies and the NCAA have implemented all these strategies of CPR in case of SCA in athletes [10, 24].

Reviewing the images and videos on the internet witnessing non-traumatic SCAs in athletes in action, it was most astonishing to observe that not a single team member or bystander started immediate cardiac massage, but instead were calling for help or tried to open the airway of the victim. Bystanders, including medical staff and teammates, were probably not to blame for not immediately recognising this lethal event. (In one case, however, three doctors responsible for the resuscitation procedure were accused of failing to treat the soccer player correctly and not using an AED. They were sentenced to prison up to one year being found guilty of manslaughter). The unexpected rare SCA came as a complete surprise, and most bystanders missed the moment of syncope because they were following the match—i. e. the ball. In addition, bystanders lost valuable time by following the AB of the ABC of the CPR protocol by trying to open the airway. This was also observed in a recently published study of the phenomenon ‘tongue swallowing’ [12]. Viskin et al. analysed 29 videos of syncope and SCA in athletes during sports activity—of which two athletes survived and three died in the most recent period of 2015–2017—obtained from the internet, and found that bystanders probably thought that the cause of the unconsciousness was the phenomenon of ‘tongue swallowing’ [12]. In the attempt to open the airway, the bystanders probably did not recognise the existence of ventricular tachycardia/ventricular fibrillation and valuable minutes went by without cardiac massage and/or defibrillation [12]. It is also hard to believe that an athlete would swallow his tongue during athletic activity as a cause of a non-traumatic SCA.

To date, there are no descriptions in the resuscitation guidelines on early recognition of SCA other than a non-responding and non-breathing or agonal breathing victim [4, 10, 13]. It appears that this description of early recognition is not optimal for bystanders to recognise non-traumatic SCA in athletes and to start CPR with cardiac massage [4, 10, 13].

### Recognising sudden cardiac arrest during sports activity

Reviewing the images and videos of some well-known cases of SCA—MVF (2003), MF (2004), AP (2007), FM (2012), PM (2012), AN (2017)—but also of camera-witnessed SCA on the internet of unknown adolescent and adult athletes participating at a lower level help us to recognise the non-traumatic syncope on the field. The, often, asymptomatic athlete participates normally in his athletic activity. All of a sudden, a loss of consciousness occurs. This can be initiated by a sustained ventricular tachycardia or ventricular fibrillation. Some athletes start leaning on their knees and all of a sudden lose their upright position failing their effort to sit down (Table 1). They fall over backwards on the ground, bounce one or more times and become unresponsive with their arms spread wide or lying alongside their body. Other athletes suddenly ‘drop dead’. They fall face down and are immediately unresponsive. The situation in which the athlete leans forward on his knees and fails to sit down is probably due to a rapid ventricular tachycardia with loss of sufficient cardiac output. After the athlete collapses to the ground and becomes unresponsive, this ventricular tachycardia becomes a pulseless ventricular tachycardia and/or deteriorates into ventricular fibrillation. The situation in which the athlete ‘drops dead’ probably reflects the onset and continuation of ventricular fibrillation. When the circulation is not restored immediately by cardiac massage, the pupils of the athlete’s eyes have a fixed gaze, frequently upward.

### Future directions

We would like to propose that a large number of the people participating in athletic activity at schools, colleges, universities or in other organised or non-organised athletic activities—i. e. athletes, team players, referees, coaches/trainers, paramedical/medical personnel, teachers, spectators—should learn how to rescue an athlete when an unexpected life-threatening situation occurs, by early recognition and the immediate start of cardiac massage. They should know how to perform BLS and how to use an AED. The AED equipment should be installed in places such as clubhouses, schools and universities. The athlete needs to be protected from SCA not only by primary prevention—i. e. eligibility screening—but also by teaching all those surrounding the athlete how they can recognise an SCA early and immediately start cardiac massage to restore circulation followed by early defibrillation with an AED. It would help if resuscitation councils describe specific recommendations for CPR in athletes suffering SCAs during sports activity.

## Conclusion

Non-traumatic syncope and SCA in athletes can be recognised by an unexpected sudden dizziness followed by loss of the upright position, loss of normal breathing and eyes wide open with fixed pupils, as seen in all six SCAs in the athletes on the soccer field. The athlete should be placed in a supine position and immediate cardiac massage should be started, followed by the necessary steps to restore normal cardiac rhythm as soon as possible.

The international resuscitation councils should include special guidelines for non-traumatic SCA in athletes during sports activity, emphasising the required knowledge for early recognition of SCA and successful resuscitation of the athlete.
